# Associations of habitual sedentary time with executive functioning and short-term memory in 7th and 8th grade adolescents

**DOI:** 10.1186/s12889-024-18014-x

**Published:** 2024-02-16

**Authors:** Veerle Van Oeckel, Louise Poppe, Benedicte Deforche, Ruben Brondeel, Marijke Miatton, Maïté Verloigne

**Affiliations:** 1 Department of Public Health and Primary Care, Ghent University, Corneel Heymanslaan 10, 9000 Ghent, Belgium; 2 Department of Movement and Sport Sciences, Vrije Universiteit Brussel, Pleinlaan 2, 1050 Brussels, Belgium; 3 Department of Epidemiology and Public Health, Sciensano, Juliette Wytsmanstraat 14, 1050 Brussels, Belgium; 4 Department of Neurology, Ghent University Hospital, Corneel Heymanslaan 10, 9000 Ghent, Belgium; 5 Department of Head and Skin, Ghent University, Corneel Heymanslaan 10, 9000 Ghent, Belgium

**Keywords:** Adolescent, Sedentary behaviour, Cognition, Executive function, Short-term memory

## Abstract

**Background:**

While there is increasing evidence for negative physical health consequences of high volumes of sedentary time and prolonged sedentary time in adolescents, the association with cognition is less clear. This study investigated the association of volumes of habitual sedentary time and prolonged sedentary time with executive functions and short-term memory in adolescents.

**Methods:**

This study has a cross-sectional observational study design. Volumes of sedentary time and prolonged sedentary time (accumulated sedentary time spent in bouts of  ≥ 30 min) were measured using the Axivity AX3 accelerometer. Six cognitive functions (spatial and verbal short-term memory; and working memory, visuospatial working memory, response inhibition and planning as executive functions) were measured using six validated cognitive assessments. Data were analysed using generalised linear models.

**Results:**

Data of 119 adolescents were analysed (49% boys, 13.4 ± 0.6 year). No evidence for an association of volumes of sedentary time and prolonged sedentary time with spatial and verbal short-term memory, working memory, and visuospatial working memory was found. Volumes of sedentary time and prolonged sedentary time were significantly related to planning. One hour more sedentary time or prolonged sedentary time per day was associated with respectively on average 17.7% (95% C.I.: 3.5–29.7%) and 12.1% (95% C.I.: 3.9–19.6%) lower scores on the planning task.

**Conclusions:**

No evidence was found for an association of volumes of habitual sedentary time and prolonged sedentary time with short-term memory and executive functions, except for planning. Furthermore, the context of sedentary activities could be an important confounder in the association of sedentary time and prolonged sedentary time with cognition among adolescents. Future research should therefore collect data on the context of sedentary activities.

**Trial registration:**

This study was registered at ClinicalTrials.gov in January 2020 (NCT04327414; released on March 11, 2020).

**Supplementary Information:**

The online version contains supplementary material available at 10.1186/s12889-024-18014-x.

## Background

Sedentary behaviour refers to any waking behaviour characterised by an energy expenditure ≤ 1.5 metabolic equivalents while in a sitting, reclining, or lying posture [1]. Overall and screen-based sedentary time increase significantly during adolescence [2], with adolescents spending on average two-thirds of their waking hours sedentary [3]. Although the evidence on the negative effects of high volumes of sedentary time is less clear for adolescents compared to adults [4], several studies conclude that more sedentary time (especially time spent in screen behaviours that are not related to school) adversely affects adolescents’ physical, mental, and social health [5]. Not only high volumes of sedentary time, but also prolonged bouts of sedentary time (or “prolonged sedentary time”) could be detrimental [6, 7].

While increasing evidence points to the negative physical health consequences of too much and prolonged sedentary time in adolescents, the association with cognition has been investigated less frequently [5]. The American Psychological Association defines cognition as all forms of knowing and awareness, such as perceiving, conceiving, remembering, reasoning, judging, imagining, and problem solving. Preliminary evidence among the general population links high volumes of sedentary time and prolonged sedentary time to decreases in brain and cognitive health through several physiological pathways involving e.g. the glucose and lipid metabolism, peripheral vascular functions, and pro-inflammatory effects [8, 9]. As adolescence is an important phase for brain maturation and cognitive development [10], and proper cognitive functioning is important for adequate academic performance [11], investigating the association of habitual sedentary time and prolonged sedentary time with general cognitive functioning in adolescents is important.

In this study, we consider cognitive functioning as an umbrella term for all cognitive subdomains. Among these cognitive functions, we distinguish executive functions and short-term memory, as reflected in Baddeley's human working memory model [12]. This model states that there is a “central executive”, what we operationalise in our study as the executive functions (i.e. a set of cognitive skills controlling complex goal-directed behaviour (e.g. working memory, inhibitory control, and planning performance) [13]). This “central executive” controls the systems that store information for a short period of time, which we operationalise in this study as the short-term memory (i.e. the capacity of the human mind to hold a limited amount of information in a very accessible state temporarily [14]).

Some studies have already investigated the relation of device-measured sedentary time and prolonged sedentary time with executive functioning among children and adolescents [15–21]. A systematic review synthesised evidence on the association between sedentary time and executive functions among children and adolescents (aged 5–17 years) [15]. Within all studies included in the review, eight studies measured sedentary time objectively by wearable devices. However, three studies were conducted in overweight or obese children rather than in the general population of children or adolescents, and will therefore not be discussed in the remainder of this paragraph. The evidence regarding the relation between device-measured sedentary time and executive functioning in the remaining five studies was mixed. The study of van der Niet and colleagues related higher volumes of device-measured sedentary time to a lower inhibitory control among 8- to 12-year-olds [16]. On the other hand, two studies showed that higher volumes of sedentary time were related to improved aspects of executive functioning [19, 21]. First, the study of Aadland and colleagues (conducted among 10-year-olds) related higher volumes of sedentary time to improved inhibitory control among boys and improved working memory among girls [19]. Secondly, Wickel and colleagues found that an increase in sedentary time from the age of 9 to 15 years predicted a better inhibitory control, working memory, and planning performance at the age of 15 years and that higher volumes of sedentary time at the age of 15 years were associated with a better inhibitory control, working memory, and planning performance [21]. Finally, three studies found no evidence for an association between sedentary time and one or more aspects of executive functioning [16–18]. First, the study of van der Niet and colleagues found no evidence for an association of sedentary time with working memory and planning [16]. Secondly, in the study of Fairclough and colleagues, conducted among 9- to 13-year-olds, no evidence for an association of volumes of sedentary time with inhibitory control and spatial working memory was found [17]. Finally, the study of Syväoja and colleagues, which was conducted among 5th and 6th graders, showed no evidence for an association between sedentary time and visuospatial working memory [18]. To our knowledge, only one study has examined the association between device-measured “prolonged” sedentary time and executive functioning among children or adolescents [20]. Mazzoli and colleagues examined the association of device-measured breaks in sedentary time (which is related to less prolonged sedentary time) with inhibitory control and working memory among 6- to 8-year-olds. They found that more sit-to-stand transitions in class were related to poorer response inhibition accuracy, whereas no evidence for associations with response inhibition time or working memory was found. However, no study has previously examined the association between device-measured sedentary time or prolonged sedentary time and short-term memory among children or adolescents. The studies mentioned above showed inconsistent results and had some limitations. Studies (a) were mainly conducted among primary school children [16, 19, 20], (b) mostly used hip and/or wrist-worn accelerometers to measure sedentary time [16–19], (c) barely examined the relation with sedentary behaviour patterns (e.g. prolonged sedentary time or breaks in sedentary time) [16–19, 21], and (d) did not look at the association between device-measured sedentary time or prolonged sedentary time and short-term memory [16–21]. Given the conflicting evidence and limitations of previous research, it is important to further investigate the association of volumes of sedentary time and prolonged sedentary time with executive functions. Secondly, considering the lacking evidence, there is a need to investigate the relation of volumes of sedentary time and prolonged sedentary time with short-term memory.

Therefore, this study aimed to investigate the association of volumes of habitual sedentary time and prolonged sedentary time, measured with a thigh-worn accelerometer, with a wide range of executive functions (i.e. response inhibition, planning, working memory, visuospatial working memory) and short-term memory (verbal and spatial short-term memory) in 7th and 8th grade adolescents. Considering the mechanisms linking high volumes of sedentary time to decreases in brain and cognitive health [8, 9], we expected that higher volumes of sedentary time and more prolonged sedentary time would be associated with a lower performance of the executive functions and short-term memory.

## Methods

The ‘Strengthening the Reporting of Observational Studies in Epidemiology’ (STROBE) statement was used to report this study [22]. The checklist of items that should be included, with references where this information can be found in this paper, is available at Open Science Framework [23].

### Participants

Baseline data were used from a controlled trial evaluating the effect of a standing desk intervention on sedentary time, and executive functions and short-term memory in adolescents. A convenience sample of 22 secondary schools in East- and West-Flanders (Belgium) was contacted between November 2019 and January 2020 by e-mail and subsequently by phone to participate in the study. Six schools were enrolled in the study (school response rate = 27.3%). The Flemish education poverty indicator (based on the number of adolescents not speaking Dutch at home, having a mother with a low educational level, receiving an education allowance and living in a neighbourhood with a high level of school delay) for the six schools ranged between 0.19 and 1.17 (scale: 0–4, with a higher number indicating a higher level of poverty among adolescents in a school). The average for school year 2019–2020 was 1.06. The principal of each school selected one 7th or 8th grade class (mostly 12- to 14-year-olds) with at least 20 adolescents attending general secondary education. All adolescents in these classes were invited to participate in the study (*n* = 132). Informed consent was obtained from 122 adolescents and at least one of their parents before participation (adolescent response rate = 92.4%). Reasons for not giving consent were: absenteeism (*n* = 1), unreturned consent forms (*n* = 1) or lack of consent (*n* = 8). The study was conducted in accordance with the declaration of Helsinki, and received ethical approval from the Ghent University Hospital ethics committee (B670201938818). This study has been registered at ClinicalTrials.gov in January 2020 (NCT04327414; released on March 11, 2020).

### Study protocol

Measurements occurred on a Monday or Tuesday in February–March 2020. The time of our visit on those days depended on the schools' availability, which was always in the afternoon. All measurements were performed in a classroom, supervised by two researchers. First, adolescents completed six online cognitive tests on a desktop, laptop or tablet. Afterwards, a paper survey on demographic characteristics (sex and age) was answered. Next, adolescents were given a wrist-worn sleep tracker and an accelerometer was applied to the thigh to measure sedentary time and physical activity. Together with the accelerometer, adolescents were provided with a small paper logbook to report device removal and reattachment. The sleep trackers and accelerometers were collected on Friday (specific retrieval time depended on the schools’ availability) of the same school week. All measures are explained more detailed below.

### Measures

#### Cognition

Cognitive functions were assessed using six tasks from the Cambridge Brain Sciences test battery [24]. Four tasks evaluated executive functions (response inhibition, planning, working memory and visuospatial working memory). The two remaining tasks assessed verbal short-term memory and spatial short-term memory. Task details are presented in Table 1.

**Table 1:** The six tasks used to assess executive functioning and short-term memory

Cognitive function Definition	Task^a^	Setup and scoring of the task
**Response inhibition** Ability to deliberately inhibit dominant, automatic, or prepotent responses when necessary [25].	Double trouble	At the top of the screen, the word "red" or "blue" appeared in a red or blue colour. At the bottom of the screen the words "red" and "blue" were displayed, coloured red or blue. The colour of these three words was not necessarily congruent to the word spelt (e.g. the word ‘red’ could be coloured blue). The participants had to click on the word at the bottom on the screen that spelt the colour of the word at the top of the screen. The participants had to solve as many challenges as possible in 90 s. The final score was calculated by subtracting the incorrectly answered trials from the number of correctly answered trials.
**Planning** Ability to organize cognitive behaviour in time and space. Planning is necessary in situations where a goal must be achieved through a series of intermediate steps each of which does not necessarily lead directly towards that goal [26].	Spatial planning	Numbered spheres from one to nine were positioned on a tree-shaped frame. The participants had to place the spheres in ascending order in as few steps as possible. The puzzles became progressively harder. Participants had three minutes to solve as many puzzles as possible. The final score equalled twice the calculated minimum number of moves needed to solve the puzzle minus the number of moves the participant needed to solve the puzzle.
**Working memory** A brain system that provides temporary storage and manipulation of the information necessary for complex cognitive tasks as language comprehension, learning, and reasoning [27].	Token search	A specified number of squares was displayed at random places. The participants had to search for a green sphere (a "token") under one of these squares by clicking on the squares. When the token was found, the participant had to remember which square the token was under, as the search for the token started again, but the token could not be found under the same square twice. The participant repeated this task until a token was found under each square. Difficulty was increased or decreased by adding or removing one square depending on whether the previous level was completed correctly. The task ended after three mistakes. The final score equalled the number of squares of the highest level that was successfully completed.
**Visuospatial working memory** A brain system that provides temporary storage and manipulation of the information necessary for complex cognitive tasks as language comprehension, learning, and reasoning [27]. Visuospatial working memory focuses on information regarding relationships between objects in space.	Monkey ladder	A specified set of numbered squares was displayed at random places. After a variable period of time, the numbers disappeared from the squares. The participants then had to click on the squares in ascending numerical order. Difficulty was increased or decreased by adding or removing one numbered square depending on whether the previous level was completed correctly. The task ended after three mistakes. The score obtained equalled the sum of the number of squares of all successfully solved levels, divided by the number of completed levels.
**Spatial short-term memory** The short-term memory reflects the capacity of the human mind to hold a limited amount of information in a very accessible state temporarily [14]. Spatial short-term memory deals with the relationships between objects in space.	Spatial span	Sixteen squares, shown in a four-by-four grid, were displayed on the screen. A specified number of squares lit up one by one. Subsequently, the participant had to reproduce the sequence correctly by clicking the squares. Difficulty was increased or decreased by lightening one square more or less depending on whether the previous level was completed correctly. The task ended after three mistakes. The final score equalled the sum of the number of squares of all successfully completed levels, divided by the number of completed levels.
**Verbal short-term memory** The short-term memory reflects the capacity of the human mind to hold a limited amount of information in a very accessible state temporarily [14]. Verbal short-term memory deals with numbers or words in a specific order.	Digit span	A sequence of digits ranging from 0 to 9 were shown on the screen. Subsequently, the participant was asked to recall the sequence correctly. Difficulty was increased or decreased by adding or omitting one digit depending on whether the previous level was completed correctly. The task ended after three mistakes. The final score equalled the highest number of sequential digits that the participant accurately reproduced.

^a^Pictures retrieved from https://www.cambridgebrainsciences.com/science/tasks (accessed on Jul. 14, 2023)

Tasks were performed online (via http://www.cambridgebrainsciences.com) on individual tablets, desktops, or laptops provided by the school. The use of desktops, laptops, or tablets depended on which devices were available at school. All students in a class simultaneously completed the tasks in a fixed order, taking approximately 20 min. Alongside the digital instruction that preceded each task, a researcher explained each task before adolescents started taking the tests using screenshots from the tasks, with the verbal instruction being similar to the digital instruction. Adolescents did not conduct a practice session, as this was not available in the Dutch version of the test battery. Test completion yielded a score (score calculation details are described in Table 1), with higher scores representing a better performance. As this study examined the relation of volumes of habitual sedentary time and prolonged sedentary time with executive functioning and short-term memory, cognitive test scores were considered to represent adolescents’ general executive functioning and short-term memory. The test scores have been validated among adults in previous research [24, 28]. For example, the study of Hampshire and colleagues showed a significant bivariate correlation between the mean standardised Cambridge Brain Sciences test battery scores and the performance on the “classic” Cattell Culture Fair task (*r* = 0.65, *p* < 0.001) [24]. Another study showed that the results of the Cambridge Brain Sciences test battery were comparable to those of a standard 2–3 h (paper and pencil) neuropsychological battery (the revised Wechsler Adult Intelligence Scale) [28]. The psychometric properties of the test battery for children and adolescents are still being investigated [29]. However, the study of Laureys and colleagues previously investigated the factor structure of executive functioning using seven out of 13 test battery tasks for adolescents (12–18 years), with the factor structure consisting of four different components (working memory, shifting, inhibition, and planning) [30]. Of the six tasks included in our study, five tasks were included in the study of Laureys and colleagues (i.e. all tasks except the digit span task).

#### Sedentary time and prolonged sedentary time

Sedentary time was measured using the Axivity AX3 accelerometer (Axivity Ltd., Newcastle, UK). This is a small and light-weight triaxial accelerometer (23 × 32.5 × 7.6 mm, 15 g). Devices were initialized at a sampling frequency of 25 Hz and a measurement range of ± 8 g using OmGui software (version 1.0.0.43; Open Movement, Newcastle University, UK). The Axivity AX3 was attached to the anterior midline of the right thigh with 3M™ Tegaderm Transparent Film Roll (3M, St. Paul, MN, USA). Accelerometers were worn during four or five school days (from Monday or Tuesday until Friday) using a 24-h protocol. Since the standing desk intervention study aimed to reduce volumes of sedentary time and prolonged sedentary time in the classroom, data were only collected at school days.

Data were downloaded using OmGui and subsequently read and calibrated in R (version 1.11–0; R Development Core Team, 2010) with the “GGIR: raw accelerometer data analysis” package (version 2.3–0) [31]. Non-wear time was detected using GGIR when on at least 1 axis, the standard deviation of the raw accelerometer data was < 13 milligravity units (mg) and the range of the data was < 150 mg during 60 min blocks, evaluated per 15 min. In addition, non-wear periods reported in the logbook were manually entered as non-wear time. Data during sleep time, measured by the Fitbit Charge 3 (see below), were excluded.

Axivity data were processed using a validated algorithm by which six types of activities (i.e. sitting, lying, standing, walking, cycling, and running) were detected in bouts of 5 s and expressed in min per total waking day [32]. Based on the 5 s bouts of sedentary activities (sitting and lying), total sedentary time and accumulated sedentary time spent in bouts ≥ 30 min were determined. A sedentary bout was defined as a period of uninterrupted sitting or lying without allowing any tolerance. The accumulated sedentary time spent in bouts ≥ 30 min was used as a proxy of prolonged sedentary time. Total sedentary time and sedentary time accumulated in bouts ≥ 30 min were standardised to a nine-hour day following the methodology described by Katapally and Muhajarine to take into account variations in accelerometer wear time [33]. As in previous research, adolescents’ data were only included in the analyses if at least two weekdays with a minimum of nine hours wear time per waking day were registered [34]. Because a valid day had to count at least nine hours wear time, data from the first and last day of measurement were often excluded. Average sedentary time per day and average accumulated sedentary time spent in bouts ≥ 30 min per day served as proxies for adolescents’ habitual sedentary time and prolonged sedentary time.

#### Covariates

Physical activity and sleep duration were included as covariates in our statistical analyses (see below) considering their influence on sedentary time and cognitive performance [35].

The Euclidean Norm Minus One (ENMO) was calculated from Axivity AX3 data as a measure for the volume of physical activity. The ENMO metric summarizes triaxial accelerometer data by taking the vector magnitude of the acceleration on the three axes at each observation (i.e. each 0.04 s); then extracting 1 g to accommodate for gravitation, rounding potential negative values to 0, and multiplying it by 1000 [36].

Sleep duration was measured with a Fitbit Charge 3 (Fitbit Inc., San Francisco, CA, USA), a wrist-worn activity tracker with a triaxial accelerometer and heart rate monitor. Fitbits were worn from Monday or Tuesday to Friday during the night. They were synchronised using the Fitbit app (Fitbit Inc., San Francisco, CA, USA), from which sleep onset and offset were extracted. Sleep onsets between 8 p.m. and 2 a.m. and sleep offsets between 5 a.m. and 9 a.m. were considered valid. Sleep duration was calculated as the time between sleep onset and offset. The validity of the Fitbit Charge 3 has not yet been determined. However, two studies measured the performance of the Fitbit Charge HR, which is an older model compared to the Fitbit Charge 3, in children and adolescents [37, 38]. The Fitbit Charge HR is the first Fitbit with multi-sensor capability, which is still used in updated versions like the Charge 3. Both studies showed an adequate sensitivity (97.0% and 95.7% respectively) of the Fitbit Charge HR in detecting sleep compared to polysomnography.

### Statistical analyses

Descriptive statistics were computed. Next, a Pearson correlation coefficient was calculated to assess multicollinearity between time spent sedentary and sedentary time spent in bouts ≥ 30 min. Multicollinearity was assumed when Pearson's r ≥ 0.6. As there was multicollinearity (*r* = 0.66), different models were created for (1) time spent sedentary and (2) sedentary time spent in bouts ≥ 30 min per cognitive task (scores for (1) double trouble, (2) spatial planning, (3) token search, (4) monkey ladder, (5) spatial span, (6) digit span), resulting in 12 models.

Generalised linear models were fitted using the *stats* package (version 4.0.2) and *MASS* package (version 7.3–54). Based on Akaike’s Information Criterion, a model with gaussian variance and identity link function was selected for all models except for the models predicting the scores on the spatial planning and double trouble task, where a negative binomial variance function with a log link function was selected. The estimates for the models with a gaussian variance and identity link function should be interpreted as the expected change in the dependent variable, when the independent variable increases with one unit (i.e. hour) and the other variables in the model are held constant. For example, a coefficient of 0.20 would indicate that one hour increase in sedentary time or prolonged sedentary time per day is associated with a mean increase in the score on a cognitive task of 0.20 units (in case of significance). For the models with a negative binomial variance function and a log link function, estimates were exponentiated such that they could be interpreted as a proportional difference in cognitive performance associated with a one-unit (i.e. hour) difference in sedentary time or prolonged sedentary time. For example, an exponentiated coefficient of 0.20 would correspond to "multiplying the cognitive test score by a factor of 0.20, per hour increase in sedentary time per day", which is the same as "an 80% decrease in the cognitive test score, per hour increase in sedentary time per day" (in case of significance). Next to sedentary time or sedentary time spent in bouts ≥ 30 min, the covariates "age", "sex", the "adolescents’ school", "average sleep time" and "average daily volume of physical activity" (measured as ENMO) were added to the model (considering their influence on sedentary time and cognitive performance). Next, the assumption of homoscedasticity was visually verified for all models using residuals versus fitted values plots. Outliers and influential observations were identified using the Cook's distance. As the Cook’s distance was smaller than one for all variables, no observations were omitted from the analyses. Finally, multicollinearity within the model was verified using the variance inflation factor, which had to be less than five, as was the case for all models. Although standardising model coefficients has its pitfalls [39], we also calculated standardised model coefficients for the models examining the association of volumes of sedentary time and prolonged sedentary time with executive functioning and short-term memory to enable the comparison of the model coefficients across the different models (see Supplementary file 1). P-values below 0.05 were considered statistically significant. The analyses were performed using R (version 4.0.2) (R Development Core Team, 2010). The dataset generated and analysed during the current study and the accompanying R script are available in the Open Science Framework repository [23].

## Results

Table 2 presents the descriptive statistics for adolescents with valid Axivity data (*n* = 119). It can be noticed that the mean scores for the double trouble task, measuring response inhibition, differ considerably from the norm scores. Although the researchers explained each task using screenshots from the tasks before adolescents started taking the tests and adolescents received a the digital instruction before each task, the researchers experienced that some adolescents struggled to understand or forgot the setup of one particular task, i.e. the double trouble task, which may explain this deviating value. Since the results of this test may not be representative of the sample, it was decided to exclude the double trouble task from the results.

**Table 2:** Descriptive statistics of the sample with valid Axivity data

**Demographics (*n* = 119)**							
Boys (n (%))			58 (49%)				
Age in years (mean ± SD), range			13.42 ± 0.60, 12.11 – 15.19				
**Sedentary outcomes (mean ± SD) (*n* = 119)**							
Number of valid days the Axivity was worn			2.83 ± 0.48				
Wear time Axivity in hours per day			15.24 ± 0.85				
Sedentary time in hours per day			6.51 ± 0.55				
Sedentary time spent in bouts ≥ 30 min in hours per day			3.45 ± 0.98				
**Covariates (mean ± SD)**							
Sleep time in hours per night (*n* = 109)			8.40 ± 0.65				
ENMO in milligravity units per day (*n* = 119)			26.17 ± 9.32				
**Scores cognitive tasks**^**a**^							
**Cognitive function measured (number of adolescents with valid data)**	**Sample scores**					**Norm scores for 12- to 15-yr-olds (mean ± SD)**^**b**^	
	**Boys**			**Girls**		**Boys**	**Girls**
	**Mean ± SD**	**Range**		**Mean ± SD**	**Range**		
Spatial short-term memory (*n* = 118)	5.56 ± 0.80	4 – 8		5.69 ± 1.01	3 – 8	6.34 ± 1.10	6.05 ± 1.01
Verbal short-term memory (*n* = 118)	5.37 ± 0.75	4 – 7		5.38 ± 0.86	4 – 7	6.72 ± 1.53	6.64 ± 1.52
Planning (*n* = 117)	18.14 ± 8.06	3 – 40		19.84 ± 7.71	8 – 40	21.65 ± 10.60	21.71 ± 10.36
Working memory (*n* = 118)	7.70 ± 1.58	2 – 11		7.87 ± 1.52	2 – 11	7.92 ± 2.04	7.59 ± 2.11
Visuospatial working memory (*n* = 118)	7.40 ± 1.08	5 – 10		7.31 ± 1.07	5 – 11	7.76 ± 1.13	7.53 ± 1.12
Response inhibition (*n* = 118)	14.41 ± 12.38	-5 – 42		16.39 ± 12.21	-1 – 39	28.12 ± 13.86	26.39 ± 14.07

*Abbreviations: ENMO* Euclidean Norm Minus One

^a^A higher score on a cognitive task represented a better performance. As the norm scores of the cognitive tasks are only available for boys and girls separately, descriptives are provided for boys and girls separately as well. The generally higher norm scores can be explained by the sample representing the norm scores being older (12- to 15-year-olds) than the adolescents in our sample (12- to 14-year-olds)

^b^Norm scores were derived from the core database of Cambridge Brain Sciences

The models examining the relation of volumes of sedentary time and prolonged sedentary time with the scores on the five cognitive tasks are shown in Table 3. A significant negative association was found between volumes of sedentary time and the score on the planning task (exponentiated B [95% C.I.] = 0.823 [0.703; 0.965], *p* = 0.015) as well as between volumes of prolonged sedentary time and the score on the planning task (exponentiated B [95% C.I.] = 0.879 [0.804; 0.961], *p* = 0.004) indicating that one hour more sedentary time or prolonged sedentary time per day was associated with respectively on average 17.7% (95% C.I.: 3.5–29.7%) and 12.1% (95% C.I.: 3.9–19.6%) lower scores on the planning task. No evidence for a significant association was found for volumes of sedentary time and prolonged sedentary time with (visuospatial) working memory, and spatial and verbal short-term memory.

**Table 3:** Results of the models^a^ examining the association of volumes of sedentary time and prolonged sedentary time with executive functioning and short-term memory

	Spatial short-term memory^b^			Verbal short-term memory^b^			Working memory^b^			Visuospatial working memory^b^			Planning^c^		
	*B*	*95% C.I.*	*p*	*B*	*95% C.I.*	*p*	*B*	*95% C.I.*	*p*	*B*	*95% C.I.*	*p*	*Exp. B*	*95% C.I.*	*p*
Sedentary time	0.055	-0.335; 0.445	0.784	0.024	-0.309; 0.357	0.889	-0.035	-0.629; 0.560	0.909	0.148	-0.286; 0.582	0.505	**0.823**	**0.703; 0.965**	**0.015**
Sedentary time spent in bouts ≥ 30 min	0.135	-0.087; 0.357	0.236	-0.010	-0.201; 0.180	0.915	0.318	-0.017; 0.652	0.066	0.067	-0.181; 0.316	0.597	**0.879**	**0.804; 0.961**	**0.004**

*Note: *A higher score on a cognitive task represented a better performance

*C.I.* confidence interval*, exp.* exponentiated*, ****bold*** = *p* < *0.05*

^a^Models were adjusted for age, sex, school, average sleep time and average daily volume of physical activity

^b^A model with gaussian variance and identity link function was selected

^c^A negative binomial variance function with a log link function was selected and resulting estimates were exponentiated such that they could be interpreted as a proportional difference in cognitive performance associated with a one-unit (i.e. hour) difference in sedentary time or prolonged sedentary time

## Discussion

Higher volumes of sedentary time and prolonged sedentary time were expected to be associated with lower performance of the executive functions and short-term memory among adolescents. However, significant associations were only found for volumes of sedentary time and prolonged sedentary time with planning, with higher volumes of sedentary time and prolonged sedentary time being associated with a lower planning performance.

In contrast to our significant negative association between volumes of sedentary time and planning, Wickel and colleagues found that an increase in device-measured sedentary time from the age of 9 to 15 years, as well as higher levels of device-measured sedentary time at the age of 15 were associated with a higher percentage of perfect solutions on a planning task when 15 years old [21]. A second study by van der Niet and colleagues found no evidence for an association between device-measured sedentary time and planning among 8- to 12-year-olds [16]. Although a similar task was used ("tower of London") to assess planning performance across the three studies, the scoring procedure differed, which makes it difficult to compare study results. Wickel and colleagues only looked at the percentage of perfectly solved puzzles (based on the minimum number of moves), whereas our score calculation took into account the number of moves the participant needed in relation to the predetermined minimum number of moves. Van der Niet and colleagues scored the task depending on the number of attempts needed to solve the problem (three points when the problem has been solved in one attempt, two if two attempts were needed, etc.), which led to a smaller score distribution compared to our study. This could explain why no evidence for an association was found between volumes of sedentary time and planning performance in the study of van der Niet and colleagues. Since, to our knowledge, this is the first study that examined the relationship of volumes of prolonged sedentary time with planning, there is no other literature with which to compare this result. However, the significant negative association found is consistent with our hypothesis. Currently, clear psychophysiological mechanisms explaining the association of volumes of sedentary time and prolonged sedentary time with planning are lacking.

No evidence for significant associations between volumes of sedentary time and prolonged sedentary time, and the tasks measuring (visuospatial) working memory, and spatial and verbal short-term memory was found. This could possibly be explained by the limited variability of the scores on the cognitive tests, which could be attributed to (1) the cognitive tasks or scoring procedures used, which may not be sensitive enough to measure small differences between adolescents, (2) the homogeneity of our sample, and (3) the rather small sample size. Consistent with our findings, the study of Mazzoli and colleagues, which is to our knowledge the only study evaluating the association between device-measured prolonged sedentary time and working memory, found no evidence for an association between class sit-to-stand transitions and working memory among 6- to 8-year-olds [20]. Also regarding the association of volumes of sedentary time with working memory, two previous studies showed results that were consistent with our findings. Van der Niet and colleagues found no evidence for a significant association between device-measured sedentary time and working memory [16]. Also Mazzoli and colleagues found no evidence for an association between device-measured class-time sitting and working memory [20]. However, the study of Aadland and colleagues (among 10-year-olds) related higher volumes of device-measured sedentary time to a better working memory [19], and also Wickel and colleagues found that an increase in device-measured sedentary time from the age of 9 to 15 years, as well as higher levels of device-measured sedentary time at the age of 15 were associated with a better working memory when 15 years old [21]. The different tests used to evaluate working memory could explain differences in study results. This can be related to the fact that single tasks measuring executive functions (such as working memory) are typically subject to task impurity [40]. Because executive functions operate on other cognitive processes, a single cognitive task is guided by other aspects (i.e. executive components as well as other non-executive function processes) than just the executive function the task aims to measure. This implies that one task can be used to measure different cognitive subdomains in different studies. The other way around, different tasks are being used in different studies to measure the same cognitive subdomain. Aligning cognitive function testing regarding the tasks and scoring methods used, is recommended for future studies. Lastly, it is also important to note that the study population in most studies (primary school children) [16, 19, 20] differed from our study (middle school adolescents), which might also explain conflicting study findings.

The lack of significant relations of volumes of sedentary time and prolonged sedentary time with executive functions and short-term memory in our study is unexpected given the emerging evidence for physiological mechanisms linking time spent sedentary with a lower cognitive performance [8, 9]. However, the current study and the previous studies mentioned did not consider the context of sedentary activities, which may attenuate the effect of volumes of sedentary time and prolonged sedentary time on cognition [16–19, 21]. For example, evidence shows that doing homework (which is cognitively engaging) is positively associated with cognition, whereas watching television (which is less cognitively engaging) is negatively associated with cognition [41]. Future research should therefore pay more attention to the context in which volumes of sedentary time are accumulated by combining device-based data with contextual information (e.g. collected through diaries).

More broadly, cognitive functioning in children and adolescents is a complex process influenced by many different factors (e.g. the context of sedentary activities, but also dietary intake, physical activity, sleeping habits, age, etc.), although current knowledge about those factors and how they are related to cognition is still incomplete [10, 41, 42]. The influence of sedentary time on cognition during adolescence might be limited compared to other factors influencing cognition during that life period, with the physiological pathways explaining the link between high volumes of sedentary time and prolonged sedentary time, and a lower brain and cognitive health, becoming more significant during adulthood. To investigate the average causal effect of volumes of sedentary time and prolonged sedentary time on cognition, a thorough causal diagram should be developed to identify all variables to be measured and (statistically) controlled for [43].

This study has some limitations. First, baseline data were used from a pilot intervention study, which resulted in a small sample size. Future research should include a larger sample. Secondly, the generalisability of our study results may be limited as the participating schools and adolescents were a convenience sample. The low response rate among schools (27.3%) should also be mentioned as a limitation within this regard. Furthermore, only secondary schools offering general education participated in the study. The relatively small sample size and homogeneity of our sample might also explain the limited variability in cognitive test scores compared to the norm scores. Next, we only measured volumes of sedentary time and prolonged sedentary time during weekdays, with an average of only 2.83 valid days, which might have limited (1) the variability in volumes of sedentary time and prolonged sedentary time among participants and (2) the representativeness of the measurement to reflect adolescents’ “habitual” sedentary time and prolonged sedentary time [44]. The limited variability in cognitive test scores and volumes of sedentary time and prolonged sedentary time could contribute to the mixed evidence found for associations of volumes of sedentary time and prolonged sedentary time with executive functioning and short-term memory. Another limitation was that, due to feasibility reasons, no extensive standardisation procedures when administering the cognitive tests were applied (e.g. distance to the screen, noise in the classroom, etc.) as a result of which cognitive test scores may not be an accurate representation of adolescents’ overall cognitive functioning. Related to this, there was no standardisation of the device used to complete the cognitive tests (i.e. in some schools adolescents completed the tests on tablets, in other schools desktops or laptops were used). As the time it takes to tap on a screen or to click with your mouse can differ, the difference in devices used could have influenced the final test scores of tasks with a time limit (i.e. the planning task). However, complex puzzles had to be solved in the planning task, so the influence of the difference in time between tapping or clicking on the final task score is expected to be smaller than in other tests where shorter tasks follow each other more quickly. In addition to this, the study of Anwyl-Irvine and colleagues showed that there were few differences in reaction times between desktop and laptop computers [45]. The other four tasks (i.e. tasks measuring working memory, visuospatial working memory, spatial short-term memory, verbal short-term memory) did not have a time limit as these tasks ended after three mistakes, resulting in a rather limited impact of the device used. Finally, we acknowledge that multiple testing was a limitation in this study, as 12 different models were analysed, which might have increased the likelihood of a type I error. However, based on Perneger's manuscript, we decided not to apply a more stringent alpha level as cut-off for “statistical significance” [46]. A first strength of this study concerns the focus on adolescents. Furthermore, this study is the first to evaluate the association with volumes of prolonged sedentary time, next to the association with total sedentary time. Lastly, by using a thigh-worn accelerometer in combination with a data reduction approach that estimates inclination, the position of the body could be determined more precisely resulting in a more accurate estimation of sedentary time and prolonged sedentary time [47]. However, currently there is no standardised method of processing device-based sedentary behaviour measures (e.g. regarding defining the epochs, cut points, or non-wear time) [47].

## Conclusions

No evidence was found for an association of volumes of device-measured habitual sedentary time and prolonged sedentary time with short-term memory and executive functions among adolescents, except for planning. Higher volumes of sedentary time and prolonged sedentary time were associated with a lower planning performance. The activity that is performed sedentary could be more important than the sedentary time itself in the association with cognition among adolescents. In future research, data on the context of sedentary time should therefore be collected, e.g. by using diaries. In addition, future studies would benefit from aligned cognitive assessments regarding the tasks and scoring methods used.

### Supplementary Information

**Additional file: Supplementary file 1.** Standardised model coefficients for the models examining the association of volumes of sedentary time and prolonged sedentary time with executive functioning and short-term memory.

## Data Availability

The dataset generated and analysed during the current study and the accompanying R script are available in the Open Science Framework repository, 10.17605/OSF.IO/JXU9N.

## Abbreviations

Mg: Milligravity units
ENMO: Euclidean Norm Minus One
C.I.: Confidence interval
Exp.: Exponentiated
