# Supplementary material for: Associations of habitual sedentary time with executive functioning and short-term memory in 7th and 8th grade adolescents
Source: BMC Public Health. 2024 Feb 16;24:495. doi: 10.1186/s12889-024-18014-x (PMC10870470; doi:10.1186/s12889-024-18014-x)
Supplement: Supplementary file 1 — Additional file: Supplementary file 1. Standardised model coefficients for the models examining the association of volumes of sedentary time and prolonged sedentary time with executive functioning and short-term memory. [file 12889_2024_18014_MOESM1_ESM.docx]

## **Supplementary file 1: standardised model coefficients for the models examining the association of volumes of sedentary time and prolonged sedentary time with executive functioning and short-term memory**

To enable the comparison of the model coefficients across the different models, standardised model coefficients for the models examining the association of volumes of sedentary time and prolonged sedentary time with executive functioning and short-term memory were calculated (see supplementary table 1). Standardised model coefficients were calculated using the *std.coef* function from the *MuMIn* package (version 1.47.5). The argument *partial.sd* was set to false in order to multiply the model coefficients by the ratio of the standard deviations of the independent variable and the dependent variable.

| Supplementary table 1. Standardised model coefficients for the models^a^ examining the association of volumes of sedentary time and prolonged sedentary time with executive functioning and short-term memory. | | | | | |
| --- | --- | --- | --- | --- | --- |
|  | Spatial short-term memory^b^ | Verbal short-term memory^b^ | Working memory^b^ | Visuospatial working memory^b^ | Planning^c^ |
|  | *(Exponentiated) standardised B* | | | | |
| Sedentary time | 0.032 | 0.015 | -0.013 | 0.073 | 0.987 |
| Sedentary time spent in bouts ≥30 minutes | 0.139 | -0.012 | 0.214 | 0.059 | 0.984 |

*Note: A higher score on a cognitive task represented a better performance.*

*^a^ Models were adjusted for age, sex, school, average sleep time and average daily volume of physical activity.*

*^b^ A model with gaussian variance and identity link function was selected.*

*^c^ A negative binomial variance function with a log link function was selected and resulting estimates were exponentiated.*
